# Erythroderma and Bullae in a Psoriatic Patient: Navigating Diagnostic Bias

**DOI:** 10.7759/cureus.94457

**Published:** 2025-10-13

**Authors:** Julia R Nadelmann, Emily R Nadelmann, Caroline Halverstam, Benedict Wu

**Affiliations:** 1 Dermatology, Albert Einstein College of Medicine, Bronx, USA

**Keywords:** autoimmune blistering diseases, bullous pemphigoid, cognitive bias, differential diagnosis, erythroderma, psoriasis

## Abstract

Erythroderma, defined as erythema and scaling involving almost the entire body surface area, is a potentially life-threatening dermatologic condition that may be idiopathic or secondary to inflammatory diseases such as psoriasis or atopic dermatitis, drug reactions, autoimmune blistering disorders, or malignancy. Bullous pemphigoid (BP), a common autoimmune blistering disorder in older adults, rarely presents with erythroderma, and its coexistence with psoriasis is uncommon. We report the case of a 62-year-old man with long-standing psoriasis who presented with one month of progressive pruritus and erythroderma following recent furosemide use, with worsening of his eruption despite a prednisone taper prescribed at an outside urgent care. He was initially treated with cyclosporine for a presumed psoriasis flare but developed numerous tense bullae shortly after admission. Biopsies obtained at presentation demonstrated subepidermal bullae with eosinophils on hematoxylin and eosin staining, and linear IgG and C3 along the basement membrane on direct immunofluorescence, confirming BP. Discontinuation of furosemide and initiation of systemic corticosteroids, followed by adjunctive therapies, led to complete remission. This case highlights the diagnostic challenges of erythroderma and the risk of anchoring bias in patients with pre-existing dermatoses. A thorough medication history, early biopsy, and immunopathologic evaluation are essential to avoid delays in recognizing atypical presentations of BP.

## Introduction

Erythroderma is a potentially life-threatening eruption characterized by significant erythema involving over 90% of the body’s surface area [1]. Psoriasis is the most frequently reported cause of erythroderma, though it can also be idiopathic or secondary to other systemic conditions, including infection, malignancy, and drug hypersensitivity reactions [1]. Bullous pemphigoid (BP) is a prevalent autoimmune subepidermal blistering disease primarily affecting individuals over 60. Erythrodermic BP is rare, and fewer than 10 cases have been documented in the literature [2-6].

The coexistence of psoriasis and BP is extremely rare, and the pathophysiologic mechanism is postulated to be epitope spreading, a process in which an immune response initially directed against one antigen gradually extends to target other structurally related antigens [7,8]. Here, we present a case of an acute onset of erythrodermic BP in a patient with psoriasis and discuss how anchoring bias led to delayed management.

## Case presentation

**Figure 1:**
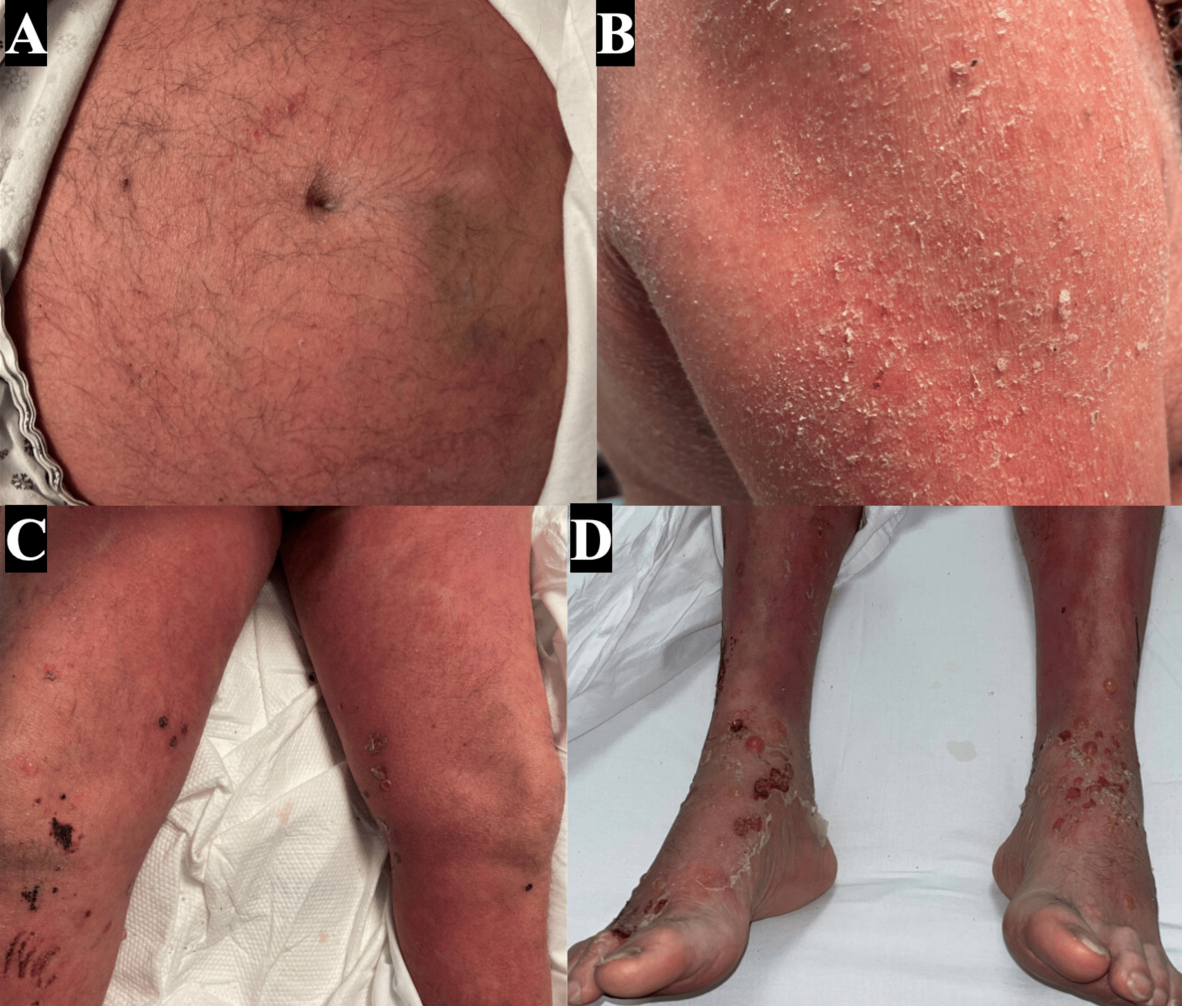
Patient presenting with diffuse erythema and scaling of his trunk and extremities (A, B), with few excoriations and crusted vesicles on his lower extremities and ankles (C, D).

A 62-year-old man with a past medical history of psoriasis presented to the hospital with a one-month history of severe pruritus and worsening eruption. Dermatology was consulted to assist with the evaluation and management of his skin findings. Physical examination revealed confluent erythema with fine scaling affecting over 90% of his body (Figure 1A, 1B). A few tense and flaccid bullae with adjacent crusted erosions were present on the acral sites (Figure 1C, 1D).

He had been taking furosemide for hypertension, though the exact duration was unclear. In addition, he had recently completed a prednisone taper for a presumed psoriasis flare, though the exact dosing and duration were not available. The complete blood count was notable for leukocytosis (13.69/nL, reference range, 3.9-10.6 /nL) and eosinophilia (2.37, reference range, 0.0-0.70/nL).

**Figure 2:**
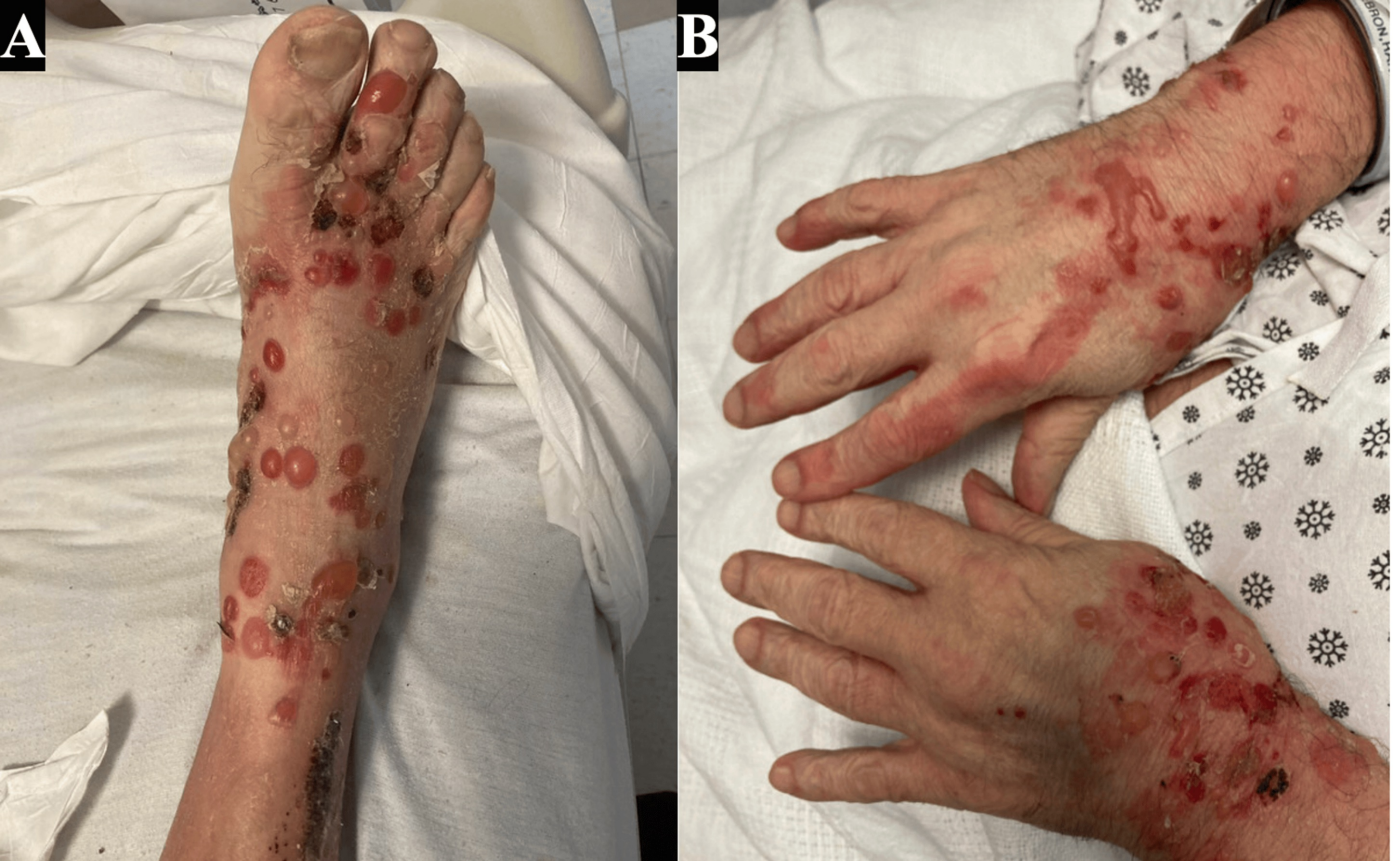
After two days of cyclosporine, numerous tense bullae appear on the trunk and acral sites.

Given the psoriasis history, he was empirically treated with cyclosporine (CSA) 2 mg/kg/day and topical triamcinolone 0.1% ointment for presumed psoriasis exacerbation triggered by steroid withdrawal. Lesional and peri-lesional punch biopsies from the left forearm were collected for histology and direct immunofluorescence (DIF). After two days of CSA, numerous tense bullae appeared on the trunk and acral sites (Figure 2).

Given the concern for BP as the cause of the erythroderma, CSA was discontinued, and intravenous methylprednisolone was started (500 mg daily for three days).

**Figure 3:**
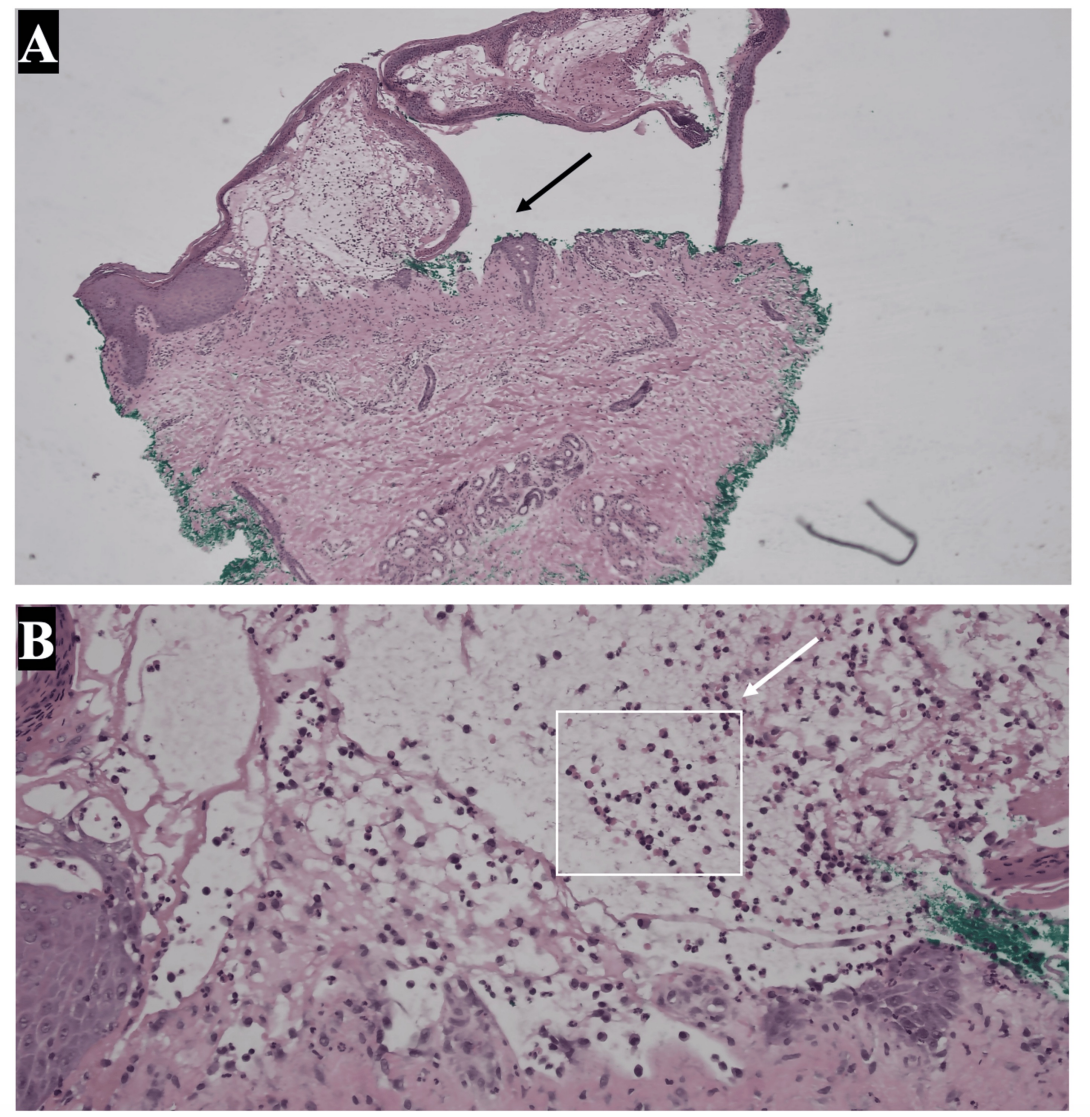
Low power (A) and high power (B) histopathologic analysis revealing subepidermal bullae with eosinophils, consistent with bullous pemphigoid.

Histopathologic analysis of the initial biopsy revealed subepidermal bullae with eosinophils (Figure 3). DIF demonstrated linear IgG and C3 deposition along the dermo-epidermal junction, confirming a diagnosis of BP. These studies were performed on the initial biopsy obtained at presentation, when erythrodermic psoriasis was still suspected. Subtle vesicles on the extremities prompted the DIF to evaluate for a blistering disorder. Additionally, anti-BPAG1 and BPAG2 antibodies were elevated to 43 (reference, <20) and >200 (reference, <20), respectively.

**Figure 4:**
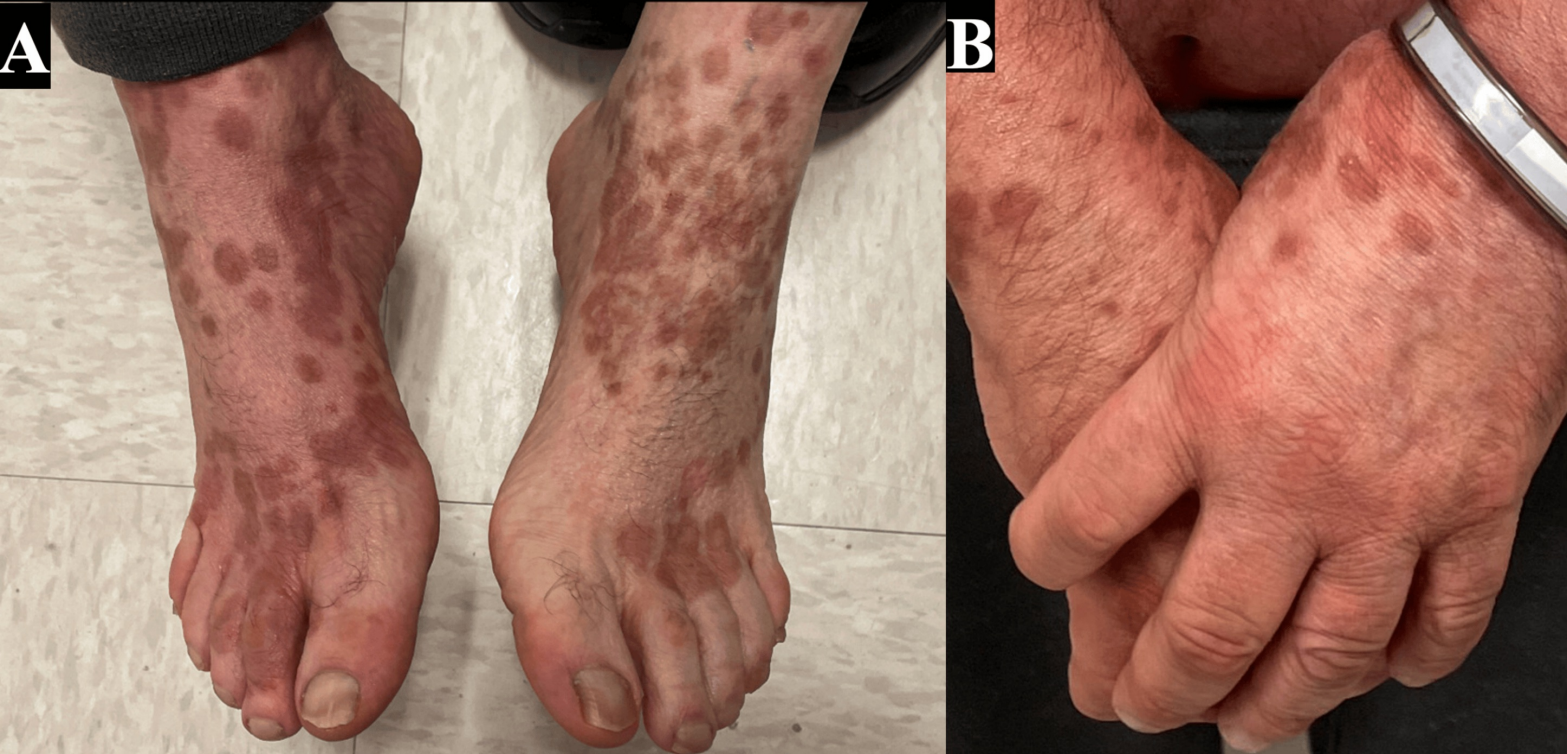
Patient at his eight-month follow-up with the resolution of erythroderma and bullae; only post-inflammatory hyperpigmentation can be seen on his dorsal feet and hands.

Given the concern for drug-induced BP, furosemide was discontinued. The patient showed significant improvement after receiving pulse intravenous methylprednisolone, followed by transitioning to prednisone 1 mg/kg/day, tapered over three months, along with doxycycline 100 mg twice daily and triamcinolone 0.1% ointment. Subsequently, he transitioned to dupilumab as maintenance therapy, administered at the standard dosing regimen for bullous pemphigoid (600 mg loading dose followed by 300 mg every two weeks), resulting in resolution of erythroderma and bullae at his eight-month follow-up visit. (Figure 4)

## Discussion

While there is a known association between BP and psoriasis, this relationship is not fully understood. The “epitope spreading” phenomenon, defined as tissue injury from chronic psoriatic inflammation leading to pathological alterations to the basement membrane zone, induces exposure to sequestered BP antigens through cross-reactivity, which is the leading theory in the association between psoriasis and BP [7,8].

Erythrodermic BP is extremely rare, with only one previously reported case in the literature involving a patient with psoriasis who was not on any medications [6]. Our case is unique because our patient has psoriasis and was exposed to a medication associated with BP, highlighting a novel clinical scenario not previously described.

In a patient with a history of psoriasis and erythroderma, it is crucial to consider their entire clinical and medication history. However, the history may lead to anchoring bias, mistakenly attributing erythroderma to psoriasis. Dermatologists should closely monitor critically ill patients, observe the evolution of the eruption, and adjust treatments as needed. This case illustrates how anchoring bias delayed appropriate treatment (prednisone instead of CSA for BP), highlighting the importance of ongoing patient evaluation for optimal outcomes. Additionally, our case underscores the diagnostic value of performing direct immunofluorescence for erythroderma, even in the absence of overt bullae, as subtle vesiculation may reveal an underlying blistering disorder.

## Conclusions

This case underscores the diagnostic challenges associated with erythroderma, particularly in distinguishing between common and rare dermatologic conditions. The coexistence of BP with psoriasis, though uncommon, highlights the importance of maintaining a broad differential diagnosis in patients presenting with worsening pruritus and erythroderma. The rapid progression to bullae formation in this patient emphasized the need for prompt histopathological and immunopathological evaluation to avoid misdiagnosis and ensure appropriate treatment.

Clinicians should remain vigilant to atypical presentations and be cautious of anchoring bias in diagnostic reasoning, particularly in complex and rapidly evolving dermatologic conditions. Early recognition and tailored therapy, including discontinuation of potential drug triggers and initiation of systemic therapies, are crucial in optimizing outcomes for such patients. This case advocates for a flexible, comprehensive approach in the assessment of erythroderma to improve diagnostic accuracy and patient care in dermatologic emergencies.
